# Initiating ivabradine during hospitalization in patients with acute heart failure: A real‐world experience in China

**DOI:** 10.1002/clc.23880

**Published:** 2022-07-23

**Authors:** Ying‐Xian Liu, Wei Chen, Xue Lin, Yan‐Lin Zhu, Jing‐Zhi Lai, Jin‐Yi Li, Xiao‐Xiao Guo, Jing Yang, Hao Qian, Yuan‐Yuan Zhu, Wei Wu, Li‐Gang Fang

**Affiliations:** ^1^ Department of Cardiology Peking Union Medical College Hospital, Chinese Academy of Medical Sciences and Peking Union Medical College Beijing P. R. China

**Keywords:** acute heart failure, ivabradine, outcome

## Abstract

**Background:**

Initiating ivabradine in acute heart failure (HF) is still controversial.

**Hypothesis:**

Ivabradine might be effective to be added in acute but hemodynamically stable HF.

**Methods:**

A retrospective cohort of hemodynamically stable acute HF patients was enrolled from January 2018 to January 2020 and followed until July 2020. The primary endpoints were all‐cause mortality and rehospitalization for HF. Secondary endpoints included heart rate (HR), cardiac function measured by New York Heart Association (NYHA) class, and left ventricular ejection fraction (LVEF) and adverse events, which were compared between patients with or without ivabradine.

**Results:**

A total of 126 patients were enrolled (50 males, median age 54 years, 81% with decompensated HF, median follow‐up of 9 months). In patients treated with ivabradine, although baseline HRs were higher than the reference group (96 vs. 80 bpm), they were comparable after 3 months; more patients tolerated high doses of β‐blockers (27% vs. 7.9%), improved to NYHA class I function (55.6% vs. 23.8%) and exhibited normal LVEFs (37.8% vs. 14.3%) than the reference group (all *p* < .05). Ivabradine was associated with a significant reduction of rehospitalization for HF than the reference group (25.4% vs.61.9%), with longer event‐free survival times (hazard ratio: 0.45, 95% confidence interval [CI]: 0.25–0.79), and was related with primary endpoints negatively (hazard ratio 0.51, 95% CI: 0.28–0.91) (all *p* < .05).

**Conclusion:**

In patients with acute but hemodynamically stable HF, ivabradine may significantly reduce HR, improve cardiac function, and reduce HF rehospitalization.

## 1 INTRODUCTION

Initiation of guideline‐directed medical therapy is encouraged as soon as patients are stabilized after hospitalization for heart failure (HF).^1^ A higher heart rate (HR) increases neurohormonal activation and metabolic demand, leading to ventricular remodeling.^2^ Given that ivabradine can be used to control HRs and decrease all‐cause rehospitalization in the “vulnerable phase” without any negative impact on blood pressure (BP) or cardiac index in chronic systolic HF,^3^, ^4^, ^5^ it is worthwhile to discuss the earliest proper time for the initiation of ivabradine.

The vulnerable phase was defined as “3 months after the date of admission due to worsening HF, which includes the period of hospitalization.”^5^ However, the evidence supporting the benefit and safety of in‐hospital initiation of ivabradine in acute HF is limited,^6^, ^7^, ^8^ there is no real‐world study on the association of in‐hospital initiation of ivabradine and outcomes in acute HF patients. Whether the initiation of ivabradine during hospitalization or early in the postdischarge period is better for acute HF patients still needs to be investigated. Additionally, the benefit in acute HF with a midrange ejection fraction (HFmrEF) or a preserved ejection fraction (HFpEF) remains unclear.

The present real‐world study aimed to (i) evaluate the impact of adding ivabradine in hemodynamically stable acute HF patients on HRs and cardiac function; (ii) assess the effects of in‐hospital initiation of ivabradine on the composite endpoint of all‐cause mortality and readmission for HF; and (iii) explore the effects of ivabradine on patients with HFmrEF and HFpEF.

## 2 METHODS

### 2.1 Population

Patients with acute HF and sinus HRs higher than 70 bpm, whether de novo or decompensated, were identified and enrolled at Peking Union Medical College Hospital between January 2018 and January 2020. The diagnostic algorithm for HF was in accordance with the European Society of Cardiology (ESC) guidelines.^4^ Patients were categorized as follows: HF with reduced left ventricular ejection fraction (LVEF) was used to designate HF patients with an LVEF less than 40% (HFrEF), HFpEF for those with an LVEF of at least 50%, and HFmrEF for those with an LVEF from 40% to 49%. The classification of diastolic function was based on the statement from the ESC.^9^ All enrolled patients had received β‐blockers (unless contraindicated) and were divided into ivabradine and reference groups according to whether or not they had started ivabradine treatment during hospitalization. Whether to initiate ivabradine or not was decided by the agreement of two cardiologists. If the consensus could not be made by the two physicians, another professor's suggestion was needed. The exclusion criteria were: ivabradine or β‐blockers initiated when hemodynamically unstable (such as during intravenous vasopressor administration); ivabradine initiated after 4 weeks of admission or discharge; serious kidney dysfunction (estimated glomerular filtration rate [eGFR] < 30 ml/min); alcoholic cardiomyopathy, hyperthyroid cardiomyopathy, moderate‐to‐severe anemia (hemoglobin <100 g/L for males and <90 g/L for females); advanced atrioventricular block; advanced malignant tumors; and ivabradine has withdrawn within 7 days of initiation.

The study was performed with the approval of the Ethics Committee of the Peking Union Medical College Hospital, which waived the requirement for informed consent because the study used anonymized data to conduct a retrospective analysis.

### 2.2 Baseline data collection

Demographic characteristics, the etiology and type of HF, comorbidities, laboratory variables, and echocardiographic reports were collected from the medical records. Indications, initial doses of ivabradine, and concomitant medication regimens were also documented. β‐blockers dosage was categorized as low, medium, and high doses (Supporting Information: Table S1). The eGFR was estimated using the Modification in Diet in Renal Disease equation.^10^

### 2.3 Definition of endpoints

Follow‐up was conducted over the phone and in outpatient clinics to assess adverse events, doses, and tolerance of ivabradine, especially when combined with β‐blockers every 3 months until July 2020. The primary endpoints were all‐cause mortality and rehospitalization for HF. The event‐free survival time (EFS) was defined as the duration from the first discharge to the occurrence of any primary endpoint. Additional adverse events recorded were cardiogenic deaths, symptomatic bradycardia, and symptomatic hypotension. The secondary endpoints were HR and the proportions of participants with an HR greater than 70 bpm 3 months after discharge and at the last visit. Other secondary endpoints were the proportion of patients receiving high doses of β‐blockers, BP, and the proportion of patients classified in the New York Heart Association (NYHA) class at 3 months and the last visit. If baseline and follow‐up echocardiography reports were available, the LVEF (Simpson's biplane technique), left ventricular end‐diastolic dimension, left ventricular end‐systolic dimension, and degree of diastolic function were also collected.

### 2.4 Statistical analysis

All analyses were performed using SPSS 19.0 (SPSS Inc.). Quantitative variables were expressed as the means ± standard deviations or medians (interquartile ranges) and were analyzed with *t*‐tests (including independent‐sample *t* and paired *t*‐test) or the Wilcoxon rank‐sum test, based on whether the data were normally or nonnormally distributed, respectively. Categorical variables were expressed as numbers and percentages and were compared using the *χ* ^2^ or Fisher's exact test. The correlation between in‐hospital length of stay and duration from admission to ivabradine initiation was tested by Spearman correlation analysis. The effects of ivabradine compared with usual care on the pre‐specified outcomes were assessed using a Cox regression model including treatment as a factor after adjustment for β‐blocker use, NYHA class, age, LVEF, and eGFR. Hazard ratios and 95% confidence intervals (CIs) were calculated. EFS was modeled with Kaplan–Meier survival analysis, and between‐treatment group comparisons were tested with the log‐rank test. A two‐tailed *p* < .05 was considered significant.

## 3 RESULTS

### 3.1 Baseline characteristics

A total of 126 patients were included in the retrospective cohort (Supporting Information: Figure S1). The median age was 54 years, and 50 of them were male. There was no difference in most baseline parameters except for a higher percentage of patients with diabetes in the reference group. The percentage of LVEF‐based classifications demonstrated no difference between patients with ivabradine and reference groups (HFrEF 66.7% [*N* = 43] vs. 63.6% [*N* = 41], HFmrEF 17.5% [*N* = 10] vs. 30.2% [*N* = 17], HFpEF 15.9% [*N* = 10] vs. 6.3% [*N* = 6], all *p* > .05, Figure 2F). The majority of included patients were optimally treated according to current guideline‐directed medical therapy (a total of 70.7% with angiotensin‐converting enzyme inhibitors, angiotensin II receptor blockers, and sacubitril/valsartan, and up to 68.3% with aldosterone). There was no difference in baseline anti‐HF medications between the ivabradine group and the reference group (Table 1). The median follow‐up was 9 months (4–16 months).

**Table 1:** Baseline characteristics of hospitalized patients with acute heart failure (HF)

	All patients	Ivabradine	Reference group	*p*
	(*N* = 126)	(*N* = 63)	(*N* = 63)	
Age (years) (median [IQR])	54.0 (36.0, 64)	45.0 (35.0, 64.0)	60.0 (47.5, 63.5)	.069
Male, *N* (%)	50 (39.7)	22 (34.9)	28 (44.4)	.363
Previous coronary revascularization, *N* (%)	31 (24.6)	13 (20.6)	18 (28.6)	.408
Previous valvular operation, *N* (%)	5 (4.0)	4 (6.3)	1 (1.6)	.361
Myocarditis, *N* (%)	9 (7.1)	7 (11.1)	2 (3.2)	.166
Hypertension, *N* (%)	51 (40.5)	23 (36.5)	28 (44.4)	.468
Diabetic mellitus, *N* (%)	47 (37.3)	17 (27.0)	30 (47.6)	.027
Chronic kidney disease, *N* (%)	37 (29.4)	14 (22.2)	23 (36.5)	.118
Dyslipidemia, *N* (%)	32 (25.4)	15 (23.8)	17 (27.0)	.838
BMI > 28 kg/m^2^, *N* (%)	25 (19.8)	15 (23.8)	10 (15.9)	.372
Smoking, *N* (%)	49 (38.9)	21 (33.3)	28 (44.4)	.273
COPD, *N* (%)	1 (0.8)	1 (1.6)	0 (0)	1
Paroxysmal atrial fibrillation, *N* (%)	18 (14.3)	11 (17.5)	7 (11.1)	.445
Hemoglobin (g/L) (mean [SD])	133 (23.4)	135 (24.2)	131 (22.5)	.056
eGFR (ml/min) (median [IQR])	74.1 (51.8, 100.5)	66.3 (47.3, 89.8)	84.4 (56.9, 109.0)	.322
ACEI/ARB, *N* (%)	67 (53.2)	28 (44.4)	39 (61.9)	.074
ARNI, *N* (%)	22 (17.5)	12 (19.0)	10 (15.9)	.814
Spironolactone, *N* (%)	86 (68.3)	40 (63.5)	46 (73.0)	.339
Oral digitalis, *N* (%)	39 (31.0)	20 (31.7)	19 (30.2)	1
Oral diuretics on discharge, *N* (%)	90 (71.4)	42 (66.7)	48 (76.2)	.324
Device therapy, *N* (%)	4 (3.2)	3 (4.8)	1 (1.6)	.611
Type of acute HF, *N* (%)				.001
De novo HF	24 (19.0)	20 (31.7)	4 (6.3)	.001
Decompensated chronic HF	102 (81.0)	43 (68.3)	59 (93.7)	.001
PASP (mmHg) (median [IQR])	34.0 (26.0, 46.0)	32.0 (25.0, 44.0)	40.0 (30.0, 49.5)	.022

Abbreviations: ACEI, angiotensin‐converting enzyme inhibitor; ARB, angiotensin receptor blocker; ARNI, angiotensin receptor‐NEP inhibitor; BMI, body mass index; COPD, chronic obstructive pulmonary disease; eGFR, estimated glomerular filtration rate; IQR, interquartile range; NEP, neprilysin; PASP, pulmonary artery systolic pressure.

### 3.2 Initiation of ivabradine

**Figure 1:**
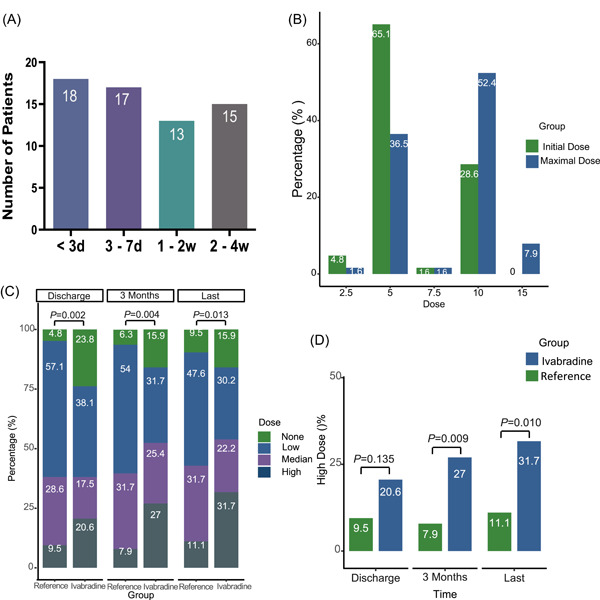
Descriptions of ivabradine and β‐blockers during the follow‐up. *χ* ^2^ and Fisher's exact test. (A) Durations from admission to adding ivabradine. (B) Initiated dose and maximal dose of ivabradine at the 3‐month follow‐up. (C) The percentage comparisons of different doses of β‐blockers between groups. (D) Percentage comparisons of HF patients with a high dose of β‐blockers at discharge, 3‐month, and last follow‐up. HF, heart failure.

The median duration from admission to initiation of ivabradine was 6 days (2–11 days). More than half of the patients (35/63) were prescribed ivabradine in the first week of admission, including 10 patients within 24 h (Figure 1A). In the group treated with ivabradine, the duration from admission to prescription was significantly correlated with in‐hospital length of stay (*R* = 0.307, *p* = .014). The most common initial daily dose was 5 mg, followed by 10 mg per day. After at least 90 days of follow‐up, the maximum daily dose of ivabradine was 10 mg for 52.4%, 5 mg for 36.5%, and 15 mg for 7.9% (Figure 1B).

### 3.3 Impact of ivabradine on titration of β‐blockers during follow‐up

During hospitalization, 76.2% of the ivabradine group and 95.2% of the reference group were treated with β‐blockers (Figure 1C). When comparing patients who did and did not receive ivabradine, 20.6% and 9.5% of them, respectively, received high doses of β‐blockers (*p* = .135, Figure 1D). After at least 3 months of follow‐up, high doses of β‐blockers were prescribed in 27% of the patients in the ivabradine group and 7.9% of the reference group (*p* = .009). At the end of follow‐up, a greater proportion of the ivabradine group patients tolerated high doses of β‐blockers than the reference group (31.7% vs. 11.1%, *p* = .01) (Figure 1D). Taken together, these results suggest a positive effect of in‐hospital addition ivabradine to help patients with acute HF titrate β‐blockers to higher doses during the vulnerable phase.

### 3.4 Comparisons of HR, BP, and cardiac function between baseline and follow‐up

**Figure 2:**
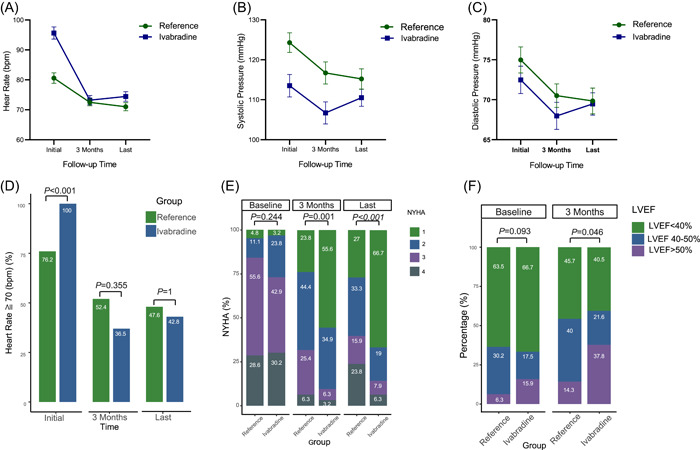
Comparisons between baseline and follow‐up. Expressed as percentages of patients with heart rates ≥ 70 bpm (A), as (median [IQR]) for heart rates (B), as mean (SD) for systolic blood pressure (C), and diastolic blood pressure (D), as distributions of NYHA classifications (E), and as distributions of HF classifications (LVEF < 40%, LVEF 40%–50%, and LVEF > 50%) (F). HF, heart failure; IQR, interquartile range; LVEF, left ventricular ejection fraction; NYHA, New York Heart Association.

Although the median baseline HR was higher in patients who received ivabradine than in the reference patients (96 vs. 80 bpm, *p* < .001), at the 3‐month follow‐up, the two groups had similar HRs (70 vs. 72 bpm, *p* = .615). Comparisons among the values at baseline, and the 3‐month follow‐up indicated a greater decrease in HR in the patients who received ivabradine than in the reference group (Supporting Information: Table S2, Figure 2A).

After 3 months of follow‐up, both systolic and diastolic BP were reduced in the reference group but not in patients treated with ivabradine. The same applied at the end of follow‐up (Supporting Information: Table S2, Figure 2B,C).

Although the baseline HR was higher than 70 bpm in all the patients who received ivabradine, 63.5% of them reached the target HR after 3 months. In contrast, 23.8% of the reference group had already met the target HR (<70 bpm) at discharge after in‐hospital up‐titration of β‐blockers, and after 3 months of usual care, 47.6% had reached the target HR. At the end of follow‐up, the target HR had been reached by 57.2% of patients treated with ivabradine and 52.4% of the reference group (Figure 2D).

Cardiac function was comparable between two groups at baseline (Figure 2E,F), but improved more over time in ivabradine group than in reference group (3‐month percentage of NYHA Class I: 55.6% vs. 23.8%, *p* = .001; at last follow‐up: 66.7% vs. 27%, *p* < .001) (Figure 2E). Meanwhile, for those patients with follow‐up echocardiographic reports (*N* = 72), although there was no difference in the baseline, the LVEF at 3 months was significantly higher in the ivabradine group than in the reference group (45.5% vs. 39.6%, *p* = .046, Supporting Information: Table S3), and a greater proportion of patients in ivabradine group achieved the target LVEF improvement (>50%) (Figure 2F).

### 3.5 Subgroup analysis of patients with HFmrEF and HFpEF

In addition to the promising results on LVEF improvement, we also explored other benefits of ivabradine on patients with normal or nearly normal LVEF. Notably, in patients with HFmrEF and HFpEF, although the group treated with ivabradine demonstrated a significantly greater HR than the reference group at baseline (101 vs. 81 bpm, *p* < .001), the HR became comparable after 3 months (68 vs. 71 bpm) and at the end of follow‐up (70 vs. 68 bpm) between patients with or without ivabradine (Supporting Information: Table S4). And a higher percentage of patients improved to NYHA Class I cardiac function than the reference group at the end of follow‐up (65.0% vs. 28.6%, *p* = .029) (Supporting Information: Table S4).

### 3.6 Primary outcomes

**Figure 3:**
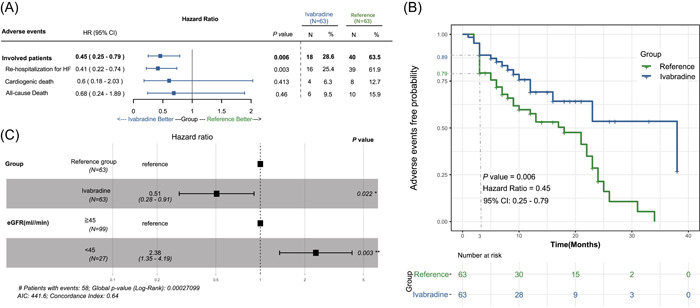
Outcome analyses of hospitalized patients with acute heart failure (HF). Multivariate Cox regression and Kaplan–Meier survival analysis. Less rehospitalization for HF (A), longer event‐free survival time (B), and reduced risk of primary outcomes (C) in the group with ivabradine. AIC, Akaike information criterion; CI, confidence interval; eGFR, estimated glomerular filtration rate.

During the follow‐up, 58 patients suffered from a total of 76 events (Supporting Information: Table S5 and Figure 3A). The overall mortality was 16/126, with 6 deaths in the ivabradine group and 10 in the reference group. Twelve were cardiogenic deaths, and three occurred within 3 months after discharge. The proportions of patients with symptomatic bradycardia and symptomatic hypotension were low (two patients in the ivabradine group and three patients in the reference group). Notably, 25.4% of the patients treated with ivabradine and 61.9% of the reference group were rehospitalized for HF (*p* < .001). Further multivariate Cox regression analysis also revealed that the benefit associated with ivabradine was mainly attributable to a reduction in rehospitalization for HF (hazard ratio: 0.41, 95% CI: 0.22–0.74, *p* = .003) (Figure 3A). Kaplan–Meier analysis yielded similar results, patients in the ivabradine group had longer EFS times than those in the reference group (hazard ratio: 0.45, 95% CI: 0.25–0.79, *p* = .006) (Figure 3B). Considering all‐cause death and rehospitalization for HF as adverse events, among the baseline parameters in the Cox regression analysis, HR, age, and eGFR < 45 ml/min were potential risk factors for adverse events, and treatment with ivabradine was a protective factor against adverse events (Supporting Information: Figure S2). In the multivariate Cox regression analysis, regardless of renal function, ivabradine could prevent patients with acute HF from developing the primary endpoints (Figure 3C).

## 4 DISCUSSION

This study demonstrated that (i) in‐hospital initiation of ivabradine combined with titration of β‐blockers in hemodynamically stable patients with acute HF significantly improved HR and cardiac function without significant change in BP; (ii) adding ivabradine before discharge was associated with a significant decrease in the risk of all‐cause mortality and hospitalization for worsening HF during and after the vulnerable phase; and (iii) HF patients with an LVEF higher than 40% also benefited from ivabradine with regard to an improvement in HR and NYHA cardiac function class (graphical abstracts).

### 4.1 Initiation of ivabradine

The most suitable timing for the initiation of ivabradine in patients with acute HF might be the period right after clinical stabilization. A rapid reduction of HR in haemodynamic unstable status may have negative effects on cardiac output, leading to organ failure or circulatory collapse. The currently acceptable timing for the initiation of ivabradine is after discharge according to the SHIFT study.^3^, ^5^ However, the CONSTATHE‐DHF study declared the effect of ivabradine on controlling HR and improving cardiac systolic functions in hospitalized patients with acute HFrEF without hypoperfusion or hemodynamic deterioration.^11^ The ETHIC‐AHF study also involved patients with acute but stable systolic HF within 24–48 h after admission, it showed that adding ivabradine not only helps more patients achieve the target HR but also improved the LVEF and decreased the BNP after 4 months of follow‐up.^7^ Although a few studies showed an HR‐controlling effect and favorable tolerability of ivabradine within the period of clinical deterioration,^12^, ^13^ a recent meta‐analysis demonstrated that the addition of ivabradine within 48 h of admission had no effect on major adverse cardiovascular events (MACEs).^14^ It indicated that in patients with cardiac shock or who were still pumping with vasopressors, it might be too early to add ivabradine. Herein, the initiation of ivabradine after hemodynamic stability and before discharge might be more beneficial to patients who were admitted for acute HF.

### 4.2 Impact of ivabradine on outcomes

Beyond symptom control, it has been demonstrated that the postdischarge administration of ivabradine can result in a better prognosis of chronic HFrEF.^3^, ^15^, ^16^ However, whether the predischarge administration of ivabradine could prevent patients with HF from adverse outcomes is controversial. Ivabradine could not reduce in‐hospital mortality in patients with acute myocardial infarction and cardiogenic shock,^17^ and only reduce cardiovascular death slightly after a short follow‐up period.^7^, ^18^ However, in the Optimize Heart Failure Care Program,^19^ patients who received ivabradine in addition to β‐blockers had lower mortality and less hospitalization for HF within 1 year after discharge. Our real‐world data also demonstrated that the in‐hospital initiation of ivabradine was associated with a reduction in the occurrence of the composite endpoint of all‐cause mortality and hospitalization for HF, and the effect was more pronounced with regard to reducing rehospitalization for HF and prolonged EFS time. Hence, in stabilized HF patients shortly after an acute HF event, ivabradine shows potential benefit in reducing the likelihood of HF rehospitalization and the occurrence of the composite endpoint.

### 4.3 Impact of ivabradine on HFmrEF and HFpEF

Ivabradine might be suitable for improving symptoms of HF, even if their LVEF is higher than 40%. The potential therapeutic effect of ivabradine in HFmrEF and HFpEF is worth discussing. It is widely accepted that treating comorbidities such as hypertension and arrhythmia is more important than prescribing classic anti‐remodeling medications in patients with HFpEF.^20^ The CHARM study demonstrated that an increased HR was correlated with cardiovascular death and hospitalization for HFmrEF and HFpEF.^21^ It is reasonable to speculate that ivabradine could improve diastolic function due to its ability to reduce HR. However, the EDIFY trial showed no effect of ivabradine on diastolic functional parameters in HFpEF.^22^ In our subgroup of patients with HFmrEF and HFpEF, ivabradine did not improve the prognosis or diastolic functional parameters, either. However, HF symptoms which were evaluated by NYHA cardiac functional classifications improved significantly. This finding was consistent with a previous study that ivabradine could improve the quality of life in acute HF.^6^ As it is reported for the first time, it still needs more data to confirm.

### 4.4 Impact of ivabradine on up‐titration of β‐blockers

Higher doses of β‐blockers rather than reducing HR may have more important prognostic utility in HF.^23^ Adding ivabradine did not lead to underdosing of β‐blockers.^6^ Furthermore, ivabradine helps more patients with HF to achieve >50% of the target doses of β‐blockers.^19^, ^24^ The benefit of ivabradine to active up‐titrating β‐blockers in our study was reasonable because we noticed better NYHA cardiac function and improvement of LVEF in patients with ivabradine, which helps avoid the negative inotropic effect of β‐blockers, making patients tolerate higher dosages of β‐blockers. Meanwhile, it should be kept in mind that the more decrease of HRs in the group with ivabradine may be attributed to both higher doses of β‐blockers and ivabradine, and achieving higher doses of β‐blockers may play an important role in contributing to better outcomes in the combined strategy group. Previous studies and our data suggest that the administration of ivabradine could be a useful therapeutic strategy to achieve higher doses of β‐blockers in hospitalized patients with acute but stable HF.

### 4.5 Limitations

As this was a retrospective observational study, selection bias could not be avoided. Meanwhile, ivabradine was prescribed in a nonrandomized manner. In addition, the follow‐up period was relatively short, limiting the opportunity to investigate the benefit of maintaining ivabradine therapy with regard to the reduction in MACEs. In the future, a multicentre registered trial with a larger sample of patients with acute but stable HF is warranted to confirm our results.

## 5 CONCLUSION

We demonstrated that the initiation of ivabradine could be considered for patients with a sinus HR higher than 70 bpm, regardless of the degree of LVEF reduction, during the period between the achievement of hemodynamic stability and discharge. We expect the early administration of ivabradine to support better HR control, improved cardiac function, and fewer adverse events, especially rehospitalization for HF.

## CONFLICT OF INTEREST

The authors declare no conflict of interest.

## Supporting information

Figure S1. Study flow of inclusion, exclusion, grouping and follow‐up. BNP, B type natriuretic peptide; NT‐proBNP, N terminal pro B type natriuretic peptide.

Figure S2. Correlations of baseline parameters and primary endpoints in the univariate Cox regression analysis.

Supporting information.

Supporting information.

Supporting information.

Supporting information.

Supporting information.

Supporting information.

## Data Availability

Data sharing not applicable.
