# Supplementary figures and images for: Initiating ivabradine during hospitalization in patients with acute heart failure: A real‐world experience in China
Source: Clin Cardiol. 2022 Jul 23;45(9):928–35. doi: 10.1002/clc.23880 (PMC9451666; doi:10.1002/clc.23880)

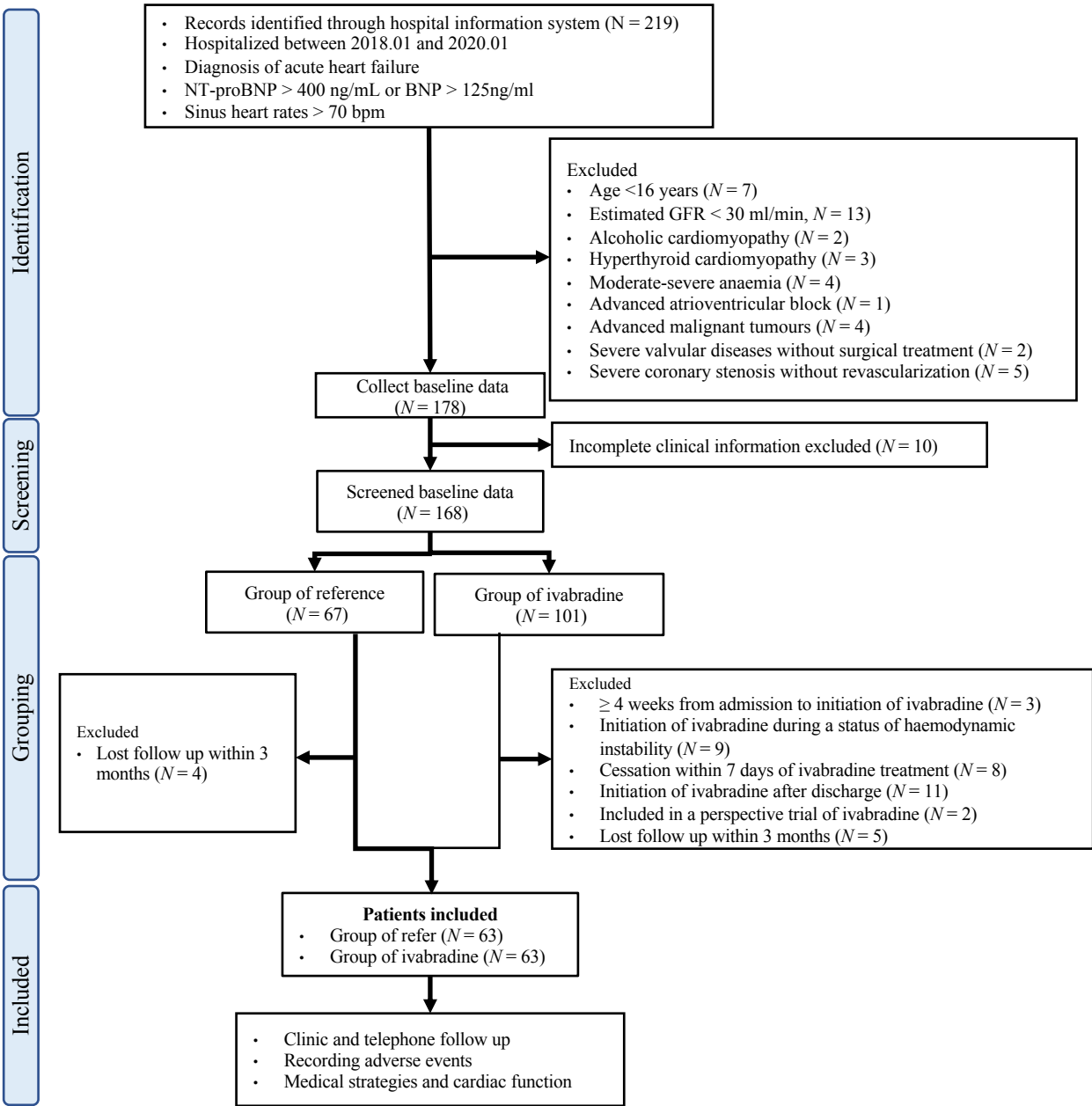

Supplement: Supplementary file 1 — Figure S1. Study flow of inclusion, exclusion, grouping and follow‐up. BNP, B type natriuretic peptide; NT‐proBNP, N terminal pro B type natriuretic peptide. [file CLC-45-928-s004.pdf]
