# Supplementary material for: Initiating ivabradine during hospitalization in patients with acute heart failure: A real‐world experience in China
Source: Clin Cardiol. 2022 Jul 23;45(9):928–35. doi: 10.1002/clc.23880 (PMC9451666; doi:10.1002/clc.23880)

Figure S2. Correlations of baseline parameters and primary endpoints in the univariate Cox regression analysis

Characteristics	*HR* (95% CI)	*P*-value
LVEDD (mm)	1 (0.97 - 1.02)	0.757
LVESD (mm)	1 (0.98 - 1.03)	0.819
LVFS	0.97 (0.93 - 1.02)	0.216
LVEF	0.99 (0.96 - 1.01)	0.284
LVEF>40%	0.61 (0.33 - 1.13)	0.115
Diastolic function grade Ⅱ-Ⅲ	1.08 (0.64 - 1.81)	0.778
Systolic BP (mmHg)	1 (0.99 - 1.01)	0.93
Diastolic BP (mmHg)	0.99 (0.98 - 1.01)	0.524
NYHA ≧Ⅲ	2.15 (0.97 - 4.76)	0.059
Age (years)	1.02 (1 - 1.04)	0.034
Group (Ivabradine)	0.45 (0.25 - 0.79)	0.006
Gender (Female)	1.64 (0.98 - 2.76)	0.06
Days of hospitalization (days)	0.98 (0.96 - 1)	0.045
Chronic kidney disease	2.65 (1.55 - 4.54)	<0.001
Diabetic mellitus	1.47 (0.88 - 2.48)	0.143
Hypertension	0.93 (0.54 - 1.61)	0.809
Dyslipidemia	0.85 (0.47 - 1.56)	0.606
Coronary artery disease	1.15 (0.68 - 1.93)	0.606
Cardiomyopathy	0.99 (0.58 - 1.68)	0.957
Myocarditis	0.66 (0.23 - 1.88)	0.439
Cardiac valvular disease	1.07 (0.26 - 4.42)	0.928
Previous valvular operation	1.23 (0.3 - 5.09)	0.775
Previous coronary revascularization	1.22 (0.69 - 2.15)	0.489
Hemoglobin(<100g/L)	1.77 (0.93 - 3.39)	0.083
Paroxysmal atrial fibrillation	0.84 (0.38 - 1.87)	0.671
ARNI	0.57 (0.23 - 1.44)	0.235
Spironolactone	0.85 (0.49 - 1.5)	0.584
Oral diuretics on discharge	1.65 (0.91 - 3)	0.1
Oral digitalis	1.37 (0.8 - 2.32)	0.251
New onset HF	1.22 (0.69 - 2.14)	0.491
Exacerbation of chronic HF	0.82 (0.47 - 1.44)	0.491
eGFR(<45 ml/min)	2.72 (1.56 - 4.74)	<0.001
Smoking	1.02 (0.59 - 1.75)	0.945
ACEI ARB	0.96 (0.56 - 1.66)	0.889
Device therapy	1.78 (0.43 - 7.41)	0.431
Decompensated chronic HF	2.74 (0.99 - 7.61)	0.053

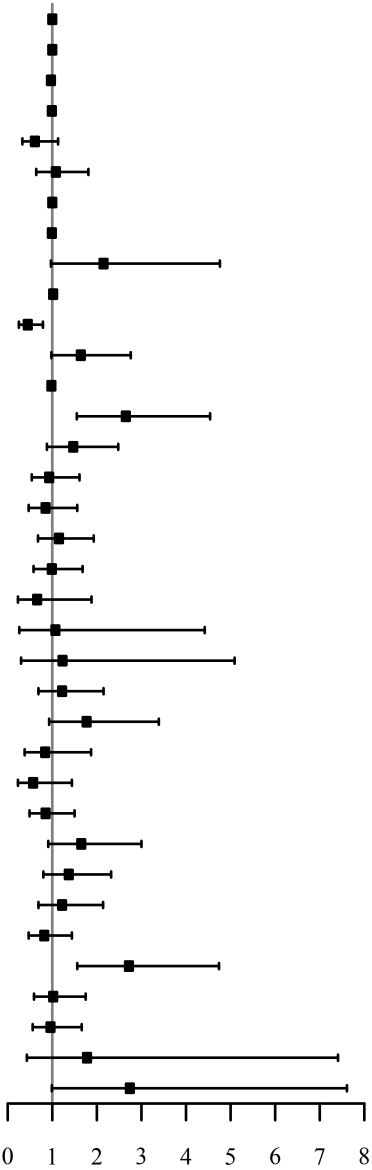

Supplement: Supplementary file 2 — Figure S2. Correlations of baseline parameters and primary endpoints in the univariate Cox regression analysis. [file CLC-45-928-s003.pdf]
