# Supplementary material for: Initiating ivabradine during hospitalization in patients with acute heart failure: A real‐world experience in China
Source: Clin Cardiol. 2022 Jul 23;45(9):928–35. doi: 10.1002/clc.23880 (PMC9451666; doi:10.1002/clc.23880)
Supplement: Supplementary file 3 — Supporting information. [file CLC-45-928-s006.docx]

| Table S1. Definitions of low, medium, and high doses in patients with β-blockers | | | |
| --- | --- | --- | --- |
|  | Low | Medium | High |
| Carvedilol (mg) | 3.12-6.25 | 12.5 | 25-50 |
| Bisoprolol (mg) | 1.25-2.5 | 3.75-5 | 7.5-10 |
| Metoprolol (mg) | 0-74 | 75-149 | 150-200 |
