# Supplementary material for: Initiating ivabradine during hospitalization in patients with acute heart failure: A real‐world experience in China
Source: Clin Cardiol. 2022 Jul 23;45(9):928–35. doi: 10.1002/clc.23880 (PMC9451666; doi:10.1002/clc.23880)
Supplement: Supplementary file 4 — Supporting information. [file CLC-45-928-s005.docx]

|  | *Ivabradine (N=63)* | | |  | *reference group (N=63)* | | |  | *P value between 2 groups*^†^ | |
| --- | --- | --- | --- | --- | --- | --- | --- | --- | --- | --- |
|  | *Baseline(N=63)* | *3 months(N=63)* | *P value* |  | *Baseline(N=63)* | *3 months (N=63)* | *P value* |  | *Baseline(N=126)* | *3 months (N=126)* |
| Heart rate (bpm) (median [IQR]) | 96.00 [86.0, 107.0] | 70.0 [66.0, 80.0] | <0.001* |  | 80.00 [71.5, 89.5] | 72.0 [66.0, 80.0] | <0.001* |  | <0.001 | 0.615 |
| Systolic BP (mmHg) (mean (SD)) | 113.5 (22.3) | 106.7 (21.8) | 0.088* |  | 124.3 (19.6) | 116.7 (22.0) | 0.041* |  | 0.004 | 0.012 |
| Diastolic BP (mmHg) (mean (SD)) | 72.5 (13.6) | 68.0 (13.37) | 0.064* |  | 75.0 (13.0) | 70.5 (11.7) | 0.044* |  | 0.291 | 0.262 |
| NYHA class, N (%) |  |  | <0.001^‡^ |  |  |  | <0.001^‡^ |  | 0.244 | 0.001 |
| 1 | 2 (3.2) | 35 (55.6) |  |  | 3 (4.8) | 15 (23.8) |  |  |  |  |
| 2 | 15 (23.8) | 22 (34.9) |  |  | 7 (11.1) | 28 (44.4) |  |  |  |  |
| 3 | 27 (42.9) | 4 (6.3) |  |  | 35 (55.6) | 16 (25.4) |  |  |  |  |
| 4 | 19 (30.2) | 2 (3.2) |  |  | 18 (28.6) | 4 (6.3) |  |  |  |  |

_Table S2. A 3-month follow up in patients with acute heart failure_

*Paired t test and †independent t test for continuous outcome measures and ‡chi-square tests for categorical outcome measures.
