# Supplementary material for: Initiating ivabradine during hospitalization in patients with acute heart failure: A real‐world experience in China
Source: Clin Cardiol. 2022 Jul 23;45(9):928–35. doi: 10.1002/clc.23880 (PMC9451666; doi:10.1002/clc.23880)
Supplement: Supplementary file 5 — Supporting information. [file CLC-45-928-s008.docx]

Table S3. Left ventricular functional comparisons between baseline and follow-up.

|  | *Ivabradine (N=63)* | | |  | *reference group (N=63)* | | |  | *P value between 2 groups*^†^ | |
| --- | --- | --- | --- | --- | --- | --- | --- | --- | --- | --- |
|  | *Baseline (N=63)* | *Follow-up Record (N=37)* | *P value* |  | *Baseline*  *(N=63)* | *Follow-up Record (N=35)* | *P value* |  | *Baseline*  *(N=126)* | *Follow-up Record (N=72)* |
| LVEDD (mm) (mean (SD)) | 60.4 (10.8) | 57.9 (10.5) | 0.269* |  | 61.7 (8.8) | 60.5 (8.8) | 0.536* |  | 0.462 | 0.257 |
| LVESD (mm) (mean (SD)) | 49.2 (11.8) | 45.1 (12.4) | 0.106* |  | 50.6 (9.3) | 48.5 (9.5) | 0.293* |  | 0.461 | 0.195 |
| LVFS (mean (SD)) | 19.2 (7.8) | 23.5 (8.3) | 0.011* |  | 18.1 (6.1) | 20.2 (6.4) | 0.113* |  | 0.385 | 0.063 |
| LVEF (mean (SD)) | 37.6 (13.0) | 45.5 (13.5) | 0.005* |  | 35.2 (9.63) | 39.6 (10.9) | 0.042* |  | 0.243 | 0.046 |
| Diastolic function grade II~III, N (%)^‡^ | 24 (38.1) | 7 (31.8) | 0.266 |  | 37 (58.7) | 15 (50.0) | 0.569 |  | 0.032 | 0.304 |
| Diastolic function grade III, N (%)^‡^ | 18 (28.6) | 3 (13.6) | 0.788 |  | 28 (44.4) | 12 (40.0) | 0.857 |  | 0.096 | 0.078 |

*Paired t test and †independent t test for continuous variable measurement and ‡chi-square tests for categorical variable measurement. LVEDD: left ventricular end-diastolic dimension; LVESD: left ventricular end-systolic dimension; LVFS: left ventricular fraction shortening; LVEF: left ventricular ejection fraction.
