# Supplementary material for: Initiating ivabradine during hospitalization in patients with acute heart failure: A real‐world experience in China
Source: Clin Cardiol. 2022 Jul 23;45(9):928–35. doi: 10.1002/clc.23880 (PMC9451666; doi:10.1002/clc.23880)
Supplement: Supplementary file 6 — Supporting information. [file CLC-45-928-s001.docx]

| Table S4. Heart rates and cardiac functional grades before and after treatment of ivabradine in patients with HFmrEF and HFpEF. | | | | | | | | | | | | | | | | | | |  |  |
| --- | --- | --- | --- | --- | --- | --- | --- | --- | --- | --- | --- | --- | --- | --- | --- | --- | --- | --- | --- | --- |
|  | **Reference group** | | | | | | |  | **Ivabradine** | | | | | | |  |  | |  |  |
|  | **Baseline** | **3 Months** | **Last** | ***P* Value**^†^ | | | |  | **Baseline** | **3 Months** | **Last** | ***P* Values** | | | |  | ***P* Values between 2 groups** | | |  |
|  | **(*N*=21)** | **(*N*=21)** | **(*N*=21)** | **Baseline vs 3 Months** | **Baseline vs Last time** | **3 Months vs Last time** | **Interation** |  | **(*N*=20)** | **(*N*=20)** | **(*N*=20)** | **Baseline vs 3 Months** | **Baseline vs Last time** | **3 Months vs Last time** | **Interation** |  | **Baseline** | **3 Months** | **Last** |  |
| **Heart.Rate** |  |  |  |  |  |  |  |  |  |  |  |  |  |  |  |  |  |  |  |  |
| Mean (SD) | 80.6 (11.6) | 71.2 (10.3) | 68.0 (12.8) | 0.008* | 0.002* | 0.372* | 0.002 |  | 101 (15.9) | 68.0 [60.0, 82.0] | 70.0 [62.3.0, 88.5] | <0.001* | <0.001* | 0.424* | <0.001 |  | <0.001† | 0.609† | 0.248† |  |
| **NYHA class**^‡^ |  |  |  |  |  |  |  |  |  |  |  |  |  |  |  |  | 1.000 | 0.410 | 0.277 |  |
| 1 | 2 (9.5%) | 5 (23.8%) | 6 (28.6%) | 0.005 | 0.029 | 0.734 | 0.057 |  | 1 (5.0%) | 9 (45.0%) | 13 (65.0%) | <0.001 | <0.001 | 1.000 | <0.001 |  | 1.0 | 0.197 | 0.029 |  |
| 2-4 | 19 (10.5%) | 16 (76.2%) | 15 (71.4%) |  |  |  |  |  | 19 (95.0%) | 11 (55.0%) | 7 (35.0%) |  |  |  |  |  |  |  |  |  |

*Paired t test and †independent t test for continuous outcome measures and ‡chi-square tests for categorical outcome measures. NYHA: New York Heart Association.
