# Supplementary material for: Initiating ivabradine during hospitalization in patients with acute heart failure: A real‐world experience in China
Source: Clin Cardiol. 2022 Jul 23;45(9):928–35. doi: 10.1002/clc.23880 (PMC9451666; doi:10.1002/clc.23880)
Supplement: Supplementary file 7 — Supporting information. [file CLC-45-928-s002.docx]

Table S5. Descriptions of adverse events in patients with acute heart failure.

|  | *Ivabradine (N=63)* | *Reference group*  *(N=63)* | *P value* |
| --- | --- | --- | --- |
| Death, N (%) | 6 (9.5) | 10 (15.9) | 0.422 |
| Death within 3 months, N (%) | 1 (1.6) | 2 (3.2) | 1 |
| Cardiogenic death, N (%) | 4 (6.3) | 8 (12.7) | 0.363 |
| Cardiogenic death within 3 months, N (%) | 0 (0.0) | 1 (1.6) | 1 |
| Re-hospitalization for HF, N (%) | 16 (25.4) | 39 (61.9) | <0.001 |
| Re-hospitalization for HF within 3 months, N (%) | 8 (12.7) | 17 (27.0) | 0.074 |
| Symptomatic bradycardia, N (%) | 1 (1.6) | 2 (3.2) | 1 |
| Symptomatic hypotension, N (%) | 1 (1.6) | 1 (1.6) | 1 |
