# Supplementary material for: Initiating ivabradine during hospitalization in patients with acute heart failure: A real‐world experience in China
Source: Clin Cardiol. 2022 Jul 23;45(9):928–35. doi: 10.1002/clc.23880 (PMC9451666; doi:10.1002/clc.23880)

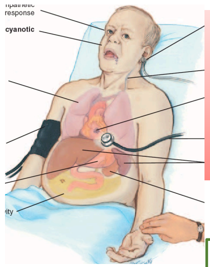

Admitted for de novo or decompensated acute HF

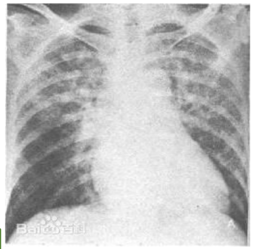

NT-proBNP > 400 ng/mL or BNP > 125ng/ml

MANAGEMENT

ARNI/ACEI/ARB
Aldosterone
Diuretics
Digitalis
Therapy for primary disease

EVALUATION

1. Stable haemodynamic status
2. Without any intravenous vasopressors
3. Initiating beta-blockers
4. Or contradictions of beta-blockers such as BP < 90/60 mmHg

Exclusion

1. Hyperdynamic cardiomyopathy such as hyperthyroid and anaemia
2. Advanced AVB

Sinus HR ≥ 70 bpm

Optimized to GDMT

1. Adding ivabradine before discharge
   - Initiated daily dose of 5-10mg
   - Maintenance in the vulnerable period
2. Titrating beta-blockers

- Shorter in-hospital length of stay
- Higher doses of beta-blockers
- Better NYHA function and LVEF
- Less HF re-hospitalization
- Longer event-free survival time

Target HR (<70 bpm) and target doses of beta-blockers

Withdrawal or decrease dose of ivabradine

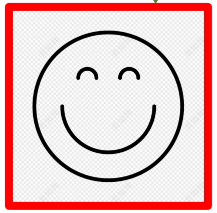

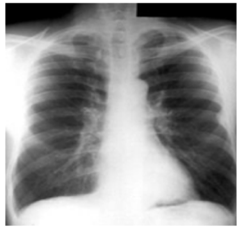

Supplement: Supplementary file 8 — Supporting information. [file CLC-45-928-s007.pdf]
